# Alternative Okazaki Fragment Ligation Pathway by DNA Ligase III

**DOI:** 10.3390/genes6020385

**Published:** 2015-06-23

**Authors:** Hiroshi Arakawa, George Iliakis

**Affiliations:** 1 IFOM-FIRC Institute of Molecular Oncology Foundation, IFOM-IEO Campus, Via Adamello 16, Milano 20139, Italy; 2 Institute of Medical Radiation Biology, University of Duisburg-Essen Medical School, Essen 45122, Germany; E-Mail: Georg.Iliakis@uk-essen.de

**Keywords:** DNA ligase 3, Lig3, DNA ligase 1, Lig1, DT40, DNA replication, Okazaki fragment, DNA repair

## Abstract

Higher eukaryotes have three types of DNA ligases: DNA ligase 1 (Lig1), DNA ligase 3 (Lig3) and DNA ligase 4 (Lig4). While Lig1 and Lig4 are present in all eukaryotes from yeast to human, Lig3 appears sporadically in evolution and is uniformly present only in vertebrates. In the classical, textbook view, Lig1 catalyzes Okazaki-fragment ligation at the DNA replication fork and the ligation steps of long-patch base-excision repair (BER), homologous recombination repair (HRR) and nucleotide excision repair (NER). Lig4 is responsible for DNA ligation at DNA double strand breaks (DSBs) by the classical, DNA-PKcs-dependent pathway of non-homologous end joining (C-NHEJ). Lig3 is implicated in a short-patch base excision repair (BER) pathway, in single strand break repair in the nucleus, and in all ligation requirements of the DNA metabolism in mitochondria. In this scenario, Lig1 and Lig4 feature as the major DNA ligases serving the most essential ligation needs of the cell, while Lig3 serves in the cell nucleus only minor repair roles. Notably, recent systematic studies in the chicken B cell line, DT40, involving constitutive and conditional knockouts of all three DNA ligases individually, as well as of combinations thereof, demonstrate that the current view must be revised. Results demonstrate that Lig1 deficient cells proliferate efficiently. Even Lig1/Lig4 double knockout cells show long-term viability and proliferate actively, demonstrating that, at least in DT40, Lig3 can perform all ligation reactions of the cellular DNA metabolism as sole DNA ligase. Indeed, in the absence of Lig1, Lig3 can efficiently support semi-conservative DNA replication via an alternative Okazaki-fragment ligation pathway. In addition, Lig3 can back up NHEJ in the absence of Lig4, and can support NER and HRR in the absence of Lig1. Supporting observations are available in less elaborate genetic models in mouse cells. Collectively, these observations raise Lig3 from a niche-ligase to a universal DNA ligase, which can potentially substitute or backup the repair and replication functions of all other DNA ligases in the cell nucleus. Thus, the old model of functionally dedicated DNA ligases is now replaced by one in which only Lig4 remains dedicated to C-NHEJ, with Lig1 and Lig3 showing an astounding functional flexibility and interchangeability for practically all nuclear ligation functions. The underlying mechanisms of Lig3 *versus* Lig1 utilization in DNA repair and replication are expected to be partly different and remain to be elucidated.

## 1. Introduction

Discontinuous, lagging strands are ligated in the process of DNA replication. During DNA repair, DNA strands need to be ligated after restoration of the DNA sequence. Thus, DNA ligation plays a key role in the final stages of DNA replication and repair. As T4 DNA ligase is often used for ligation of DNA fragments during vector construction and cloning, DNA ligation is one of the most familiar biochemical reactions in molecular biology.

Biochemically, DNA ligase makes a phosphodiester bond between the 5' end (donor) and 3' end (acceptor) of DNA strands [1,2,3]. Both eukaryotic and phage DNA ligases require ATP for ligation reaction that proceeds as follows [1,2,3]: First, ATP binds at the lysine residue in the catalytic active site of the enzyme as AMP. AMP is then transferred to a phosphate group at the 5' end of DNA. Finally, by releasing AMP, a phosphodiester bond is generated between the 5' phosphate group and 3' hydroxyl group.

While DNA ligases of eubacteria such as *E. coli* require NAD^+^ instead of ATP as a cofactor, the molecular mechanism of DNA ligation is similar among eukaryotic and bacterial DNA ligases. Indeed, six amino acids located in the catalytic center of the enzyme are well conserved during evolution among different species and phyla, including different DNA ligase families [4]. This strongly suggests that the basic mechanism of DNA ligation is well conserved during evolution.

**Figure 1:**
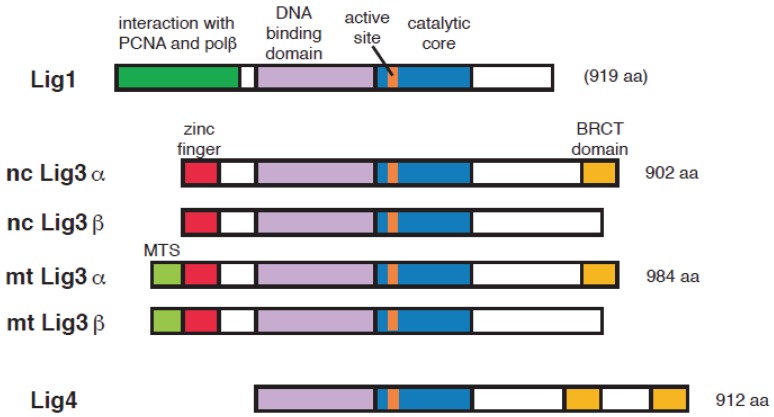
Domain structure of vertebrate DNA ligases. Nc: nuclear; mt: mitochondria.

In higher eukaryotes, the DNA ligase genes are classified in three families [1,2,3] (Figure 1). The DNA ligase 1 (Lig1) and the DNA ligase 4 (Lig4) families are present from yeast to human, and are widely conserved in the course of evolution. The DNA ligase 3 (Lig3) family is evolutionary younger and found only in vertebrates. DNA ligase 2 (Lig2) is recognized as a partial degradation product of Lig3 [5] and is currently treated as a missing number.

It is commonly thought, quasi text-book view, that Lig1 is responsible for DNA replication and long-patch base excision repair (BER); that Lig4 is responsible for the classical DNA-PKcs-dependent non-homologous end-joining (C-NHEJ); and that Lig3 works in short-patch BER, in single strand break repair, and within the mitochondria. In this current view of DNA ligase function, each family is assigned with rather specific functions in DNA metabolism. Since knockout of either Lig1 [6,7], Lig3 [8] or Lig4 [9,10,11] genes in the mouse is embryonic lethal, only limited genetic analysis has been available.

However, advanced genetic approaches can overcome this limitation and have recently generated results that challenge the view of functional dedication among the families of DNA ligases. They document an astounding functional flexibility for Lig3 that appears to be able to efficiently cover, or at least backup, the nuclear functions of both Lig1 and Lig4. Thus, a new molecular model can now be postulated that includes functional flexibility for the Lig3 family of enzymes and accommodates overlapping roles for Lig1 and Lig3 in DNA metabolism. Here, we review key findings supporting this model, as these have been obtained using powerful genetic systems based on cell lines with unique properties.

## 2. DNA Ligase Responsible for the Ligation of Okazaki Fragments

Lig1 has a central role in the ligation of Okazaki fragments during DNA replication. Lig1 is recruited to DNA replication factories via protein-protein interactions using the characteristic N-terminal regions located rather remotely from the enzyme active site [12,13] (Figure 1). The essential function of Lig1 in DNA replication is clearly shown by the lethal phenotype of *CDC9* mutation, the Lig1 homolog in S. cerevisiae [14,15,16] that has no gene corresponding to Lig3.

On the basis of these results and the above discussed postulate of functional dedication among DNA ligases, it was predicted that inactivation of the vertebrate Lig1 gene would be lethal. Indeed, two studies found that Lig1 knockout mice are embryonic lethal [6,7]. However, these independent observations received conflicting interpretations in the two papers. While Petrini *et al.*, considered that Lig1 is required for the survival and proliferation of mouse cells, Bentley *et al.*, concluded that Lig1 is not required for cell survival and proliferation, because actively growing mouse embryonic fibroblasts (MEF) could be established from early-stage embryos.

Investigators favoring the lethal phenotype of Lig1 knockout criticized the knockout strategy in the latter study, as the authors opted to delete only a small segment of the Lig1 C-terminal region, leaving intact the catalytic domain, including the enzyme catalytic center. Thus, it was argued that residual Lig1 activity could not be entirely ruled out. As apparent support for this interpretation served the observation that the established MEFs showed no sensitivity to DNA damaging agents and relatively minor defects in Okazaki fragment ligation [7,17]. As we will describe shortly, both endpoints are markedly compromised in a human cell line carrying a mutation in Lig1 gene.

The human Lig1 mutant fibroblast cell line 46BR was established from an immunodeficient patient who inherited one inactive Lig1 allele and one coding for a protein with very low enzymatic activity [18,19]. 46BR cells show no apparent growth defects, but significant defects in Okazaki fragment ligation [20,21]. 46BR cells are also hypersensitive to UV-light, in line with the function of Lig1 in NER [22]. Furthermore, 46BR cells are hypersensitive to ionizing radiation (IR), and to simple alkylating agents [7,17], suggesting a defect in long-patch BER [23]. Phenotypic differences between 46BR cells and the knockout MEFs described above may reflect the hypomorphic nature of the 46BR mutation, as well as the possibility that in 46BR cells Lig1 polypeptides expressed from the inactive allele exert dominant negative effects on partially active polypeptides.

## 3. Has Lig3 A Function in DNA Replication Outside Mitochondria?

The Lig3 gene, from a single genetic locus, can generate four different proteins with DNA ligase activity [1]. While the majority of Lig3 mRNA is translated starting from the second ATG codon on the mRNA, about 10% of translation-initiation events use the first ATG codon, that adds ~80 amino acids to the polypeptide. This endows the resulting protein with a mitochondria target sequence (MTS) that allows its transport into this DNA containing organelle, in order to cover all its DNA ligation needs [24,25] (Figure 1). Notably, in *S. cerevisiae*, this function is served by the mitochondrial form of a polypeptide encoded by the Lig1 gene. Thus, the mitochondrial DNA ligation function has been transferred from Lig1 to Lig3 during evolution from yeast to vertebrates.

In addition, a specific alternative splicing mechanism on the Lig3 mRNA replaces in some cell types the C-terminal coding exon of Lig3α by a shorter exon lacking the breast cancer susceptibility gene 1 (BRCA1) C terminus (BRCT) domain, which is required for the interaction with X-ray repair cross-complementing protein 1 (XRCC1). This alternative splicing results in the generation of a Lig3 form termed Lig3β, which is specifically found in germ cells [25,26] (Figure 1).

As mentioned above, the Lig3 gene appeared later in evolution and is found only in the vertebrate genome. However, the ancestor of Lig3 gene may have arisen earlier in evolution, as a large-scale homology search against the DNA-binding domain and catalytic core region of Lig3 gene identified Lig3 homologues in eukaryotes, including organisms that evolved before the divergence of metazoa [27]. Many of them are likely to also encode an MTS, suggesting a specific function in mitochondria. Interestingly, distribution of the Lig3 ancestor gene is spotty among divergent evolutional branches, indicating that these ancestor Lig3 genes may have been lost several times during evolution.

As Lig3 is not present in the yeast genome, it can be inferred that it does not serve essential functions. This raises the question as to whether Lig3 is actually unnecessary or non-essential in vertebrates.

Experiments designed to address this important question gave the surprising result that knockout of Lig3 gene in mouse shows an even more severe phenotype than the knockout of either Lig1 or Lig4 genes. Lig3 knockout mice show early embryonic lethality, and this lethality cannot be rescued by deletion of p53 [8]. Although it is clear that Lig3 is essential for mouse development and survival, it remained an open question whether Lig3 is essential for the survival of cells. Indeed, Lig1 deficiency is lethal in the mouse, but mouse cells can survive and proliferate without Lig1 (see above). We addressed this important question using DT40 cells.

**Figure 2:**
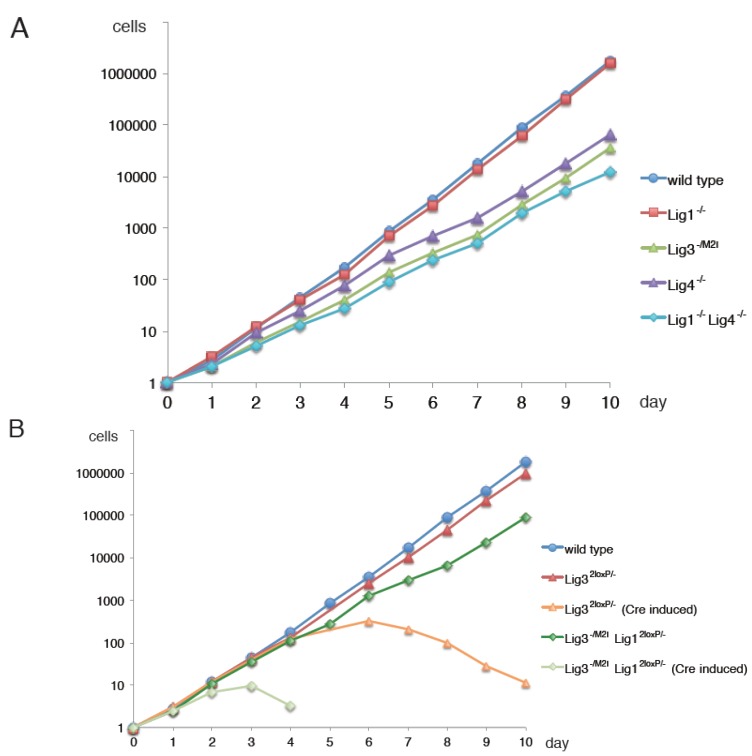
Synthetic lethality between Lig1 and nuclear Lig3. (**A**) Growth kinetics of wild-type and DNA ligase knockout DT40 cell lines; (**B**) Growth kinetics of wild-type and DNA ligase conditional knockout DT40 cell lines. *Lig1^2loxP/−^:* Lig1 conditional knockout, *Lig3^2loxP/−^:* Lig3 conditional knockout, *Lig3^−/M2I^*: Nuclear-Lig3 knockout. Adapted from [4].

The chicken B cell line DT40 shows high frequency of targeted integration [28] and has been used as a model system of gene knockout studies, especially for genes of the DNA metabolism [29]. Our groups have carried out a systematic knockout of all DNA ligase genes in DT40, to clarify the functions of Lig3 in DNA metabolism [4]. In the course of these studies, we observed in repeated trials that it was not possible to generate homozygous Lig3 gene knockouts using a conventional knockout design; in contrast it was rather straightforward to generate heterozygous Lig3 gene knockouts. We assumed that Lig3 was essential for the survival of DT40 cells, and switched to conditional knockout strategies using the Cre/lox P system [30]. Lig3 conditional-knockout DT40 cells were found to proliferate well. However, when these cells are converted to the Lig3 knockout state by Cre/lox P recombination, cell growth shows severe defects soon thereafter and cells die in about a week [4] (Figure 2B). These experiments clearly demonstrate that Lig3 is essential for DT40 cell survival.

Since dying cells are difficult to analyze genetically, the lethal phenotype of Lig3 knockout, observed in DT40 as well, was disappointing. However, we speculated that the observed lethality derives from the function of Lig3 in mitochondria (see above). To directly examine this postulate, we replaced the second start ATG (Met) codon with ATC (Ile) generating thus a gene producing the mitochondrial but not the nuclear form of Lig3 (*Lig3^−/M2I^*)—*i.e.*, a knockout specifically for the nuclear form of Lig3. Notably, this nuclear Lig3-specific DT40 mutant grows normally without showing any obvious phenotypes [4] (Figure 2A). Thus, the essential role of Lig3 resides in its function in the mitochondria DNA metabolism [31]. This characteristic is also observed in mouse cells and animals [32,33]. Thus, nuclear Lig3 is not required for the survival of cells or even the mouse.

## 4. Why is Mitochondrial Lig3 Essential?

DNA ligase activity is required for the ligation of DNA fragments during duplication of the mitochondrial genome. Mitochondria have also DNA repair capacity including BER [34], and a DNA ligase is required for this function as well.

Lethality of Lig3 DT40 knockout can be rescued by overexpression of a mitochondria specific human Lig3 gene [4]. Interestingly, even the overexpression of the yeast Lig1 homolog *CDC9* can rescue the lethality of Lig3 knockout [4]. Jasin’s group showed that lethality of Lig3 knockout mouse cells can be rescued by an MTS-tagged *E. coli* DNA ligase [33], which utilizes NAD^+^ instead of ATP as cofactor (see above). Since in mitochondria, DNA is not organized in chromatin and proliferating cell nuclear antigen (PCNA) is absent, it appears that there is great flexibility in the recruitment of practically any DNA ligase to mitochondrial DNA replication factories. This is why Lig3 knockout cells can be rescued by the expression of DNA ligases of diverse origin, provided that they can enter mitochondria.

It is possible to specifically delete mitochondrial DNA by prolonged treatment of vertebrate cells with low concentrations of ethidium bromide (rho0 cells) [35]. This is because mitochondrial DNA is less protected than genomic DNA residing in the cell nucleus. Although rho-cells need special conditions to survive, the feasibility of their generation demonstrates that mitochondria are not essential for cell viability and raises the question as to why mitochondrial DNA ligase is essential for cell survival. To date, this aspect has not been thoroughly investigated. One possibility is that the absence of the enzyme from an otherwise functional organelle elicits apoptosis, particularly in DT40 cells that are highly pro-apoptotic. In addition to aerobic respiration and ATP synthesis, mitochondria also serve as a reservoir of apoptosis-related proteins including cytochrome C [36]. When in DT40 cells mitochondrial DNA replication cannot be completed, these organelles may start collapsing, generating pro-apoptotic signals.

## 5. Alternative Okazaki-Fragment Ligation Pathway by Lig3

Although the above described experiments suggest that Lig3 is not essential for cell viability, the viability of Lig1 deficient cells also suggests that Lig3 may substitute for Lig1 under certain conditions. To find clues regarding the spectrum of physiological functions of Lig3, we opted to knockout the Lig1 gene in DT40 [4]. For added flexibility in the generation of this genetic system, we also took the strategy of conditional knockout. For this purpose, most of the Lig1 exons including the entire catalytic domain were flanked by two loxP sites. After converting to a knockout, cells of this conditional mutant proliferate actively and show no signs of stress [4] (Figure 2A). Since Lig1 is normally responsible for Okazaki fragment ligation, it was surprising that Lig1 is not required for cell growth, and that Lig1 knockout DT40 cells show no phenotype, even in the form of decreased growth rate. Indeed Lig1 deficient DT40 cells show normal maturation of Okazaki fragments [4]. Lig1 may be required, however, in certain cell types or in some stages in mouse development, as Lig1 knockout mouse shows early embryonic lethality [6,7]. However, as noted above, in MEFs Lig1 is not essential, and indeed Lig1 knockout mouse B cells also exhibit normal proliferation characteristics and normal sensitivity to a wide variety of DNA damaging agents [37]. Similar results are also obtained with DT40 cells after exposure to UV-light [38].

As a next step, we combined the nuclear-Lig3-specific knockout with the Lig1 conditional knockout [4]. When the Lig1-conditional knocked out allele is converted to knockout, cells show severe defects in DNA replication and die after a short period of time [4] (Figure 2B). In contrast, nuclear-Lig3-specific knockout, or Lig1 single knockout cells grow normally [4].

These experiments directly and convincingly demonstrate that the protein complementing Lig1 deficiency in Okazaki fragment ligation is Lig3. Because ligation of Okazaki fragments by Lig1 is hitherto considered textbook knowledge, it was rather surprising to find that a rather “underestimated” gene, Lig3, efficiently supports such a central metabolic process of the cell.

We also generated a systematic knockout of other DNA ligase genes including Lig4 in DT40 [4]. The Lig1/Lig4 double knockout DT40 mutant is also viable, and has growth characteristics comparable to those of the Lig4 single knockout mutant [4] (Figure 2A). Efficient growth of the Lig1/Lig4 double knockout mutant that only expresses Lig3 demonstrates that this ligase can efficiently serve all DNA replication functions of Lig1 in vertebrates. Structural similarities between Lig1 and Lig3 [39,40] provide a theoretical basis for the duplication of functions between these two enzymes during evolution.

Lig1 is recruited to the sites of DNA replication through its interaction with PCNA [12,13,41]. The mechanism by which Lig3 is recruited to DNA replication sites remains to be investigated. Le Chalony *et al.* reported that Lig3 and XRCC1 are required for the proliferation of Lig1-depleted mouse and human cells, and that both Lig3 and XRCC1 are retained on chromatin and accumulate at replication foci [42]. Thus, one possibility is that Lig3 may be recruited to replication factories via interaction of PCNA with XRCC1, which is also a close partner of Lig3 [43].

## 6. Lig3 as a Universal DNA Ligase

Although Lig4 knockout cells are viable, they show severe defects in NHEJ (classical NHEJ pathway), which depends on DNA-PK and Ku. However, Lig4 knockout cells still repair DSBs, albeit inefficiently and with slow kinetics using a Backup-NHEJ (B-NHEJ) pathway (also called alternative NHEJ) [44,45,46,47,48]. B-NHEJ also plays a role in the development of the immune system *in vivo*, and particularly in class switch recombination that normally relies on components of the classical NHEJ pathway [49,50]. Also, a study using mutant Rag proteins with reduced affinity for the generated DNA ends shows a potential role of B-NHEJ in V(D)J recombination in NHEJ-deficient cells and even in wild-type cells [51]. However, this “back-up” end-joining does not always fully complement NHEJ deficiency; indeed, selective deletion of Lig4 in the central nervous system generates endogenous breaks that remain unrepaired [52].

We have previously suggested that Lig3 is involved in the repair of DSB by B-NHEJ [53]. Our *Lig1^−/−^ Lig4^−/−^* mutant provides now a conclusive genetic proof for this function. Notably, this double mutant shows comparable levels of DSB repair as Lig4 single knockout cells [54] demonstrating thus the ability of Lig3 to support B-NHEJ. Interestingly, Lig4 may also play a role in B-NHEJ in the absence of Ku [31]. Furthermore, there may be functional redundancy between Lig3 and Lig1 in B-NHEJ, as B-NHEJ is still active in *Lig3^−/M2I^ Lig4^−/−^* DT40 cells [54]. Lig3 in involved in telomere maintenance and is required by cells escaping telomere-fusion-driven crisis [55].

Lig3α-Xrcc1 is a key component of BER, and of a specific sub-pathway of NER [1,56]. Lig1/Lig4 double knockout DT40 cells, which exclusively rely on Lig3 for DNA repair are also resistant to UV light [38]. Thus, Lig3 can support NER. Evidently, in contrast to *CDC9*, which is exclusively involved in excision repair, both Lig3 and Lig1 are involved in this repair pathway in vertebrate systems. Weinfeld’s group reported that Lig3 can function as a sensor for single strand break repair (SSBR) [57]. Interestingly, for this function, Lig3 does not require its BRCT domain, and does not even require interaction with Xrcc1, suggesting the presence of other key protein partners for Lig3.

## 7. Conclusions

The above reviewed results uncover impressive and previously unrecognized potential of Lig3 to participate in all aspects of DNA repair and replication (Table 1). Only a few years ago, Lig3 was considered a “niche”-ligase, which in addition to its function in the mitochondria, served the nucleus solely in a sub-pathway of BER and in SSBR. Indeed, some investigators considered its presence in the cell nucleus unnecessary. Our work in the DT40 cell system, but also work carried out in mouse and human cell systems, allows the development of a more complete view regarding ligase functions in vertebrates: Since Lig1/Lig4 double knockout DT40 cells are viable and healthy, it can be concluded that Lig3 can efficiently substitute, or at least backup, the functions of other DNA ligases in all essential aspects of the DNA metabolism. From these functions, only Lig4-mediated DSB repair is supported with lower efficiency. DNA replication, BER, NER and even HRR [58] are supported by Lig3 with efficiency practically indistinguishable from that of Lig1. We conclude that Lig3 is a major DNA ligase with a spectrum of putative activities similar to Lig1. Thus, Lig1 and Lig3 may be considered “cousins”. Importantly, the similarity in the spectrum of activities between Lig1 and Lig3 was grossly underestimated in the field. Ultimately, Lig3 may have evolved from the duplication of a functional homolog to generate a dedicated mitochondrial ligase and a back-up system for all essential nuclear Lig1 functions. The evolutionary “persistence” of this “solution” in vertebrates suggests that it confers distinct advantages to the organism.

**Table 1:** Roles of DNA ligases.

Concept	Lig1	Lig3	Lig4
classical concept	DNA replication	BER (short patch)	NHEJ
	BER (long patch)	mitochondria	V(D)J recombination
	HR		class switch recombination
	NER		
new concept	DNA replication	DNA replication	NHEJ
	BER (long patch)	BER (short patch)	V(D)J recombination
	HR	HR	class switch recombination
	NER	NER	
	B-NHEJ	B-NHEJ	
	(V(D)J recombination?)	(V(D)J recombination?)	
	(class switch recombination?)	(class switch recombination?)	
		mitochondria	

## 8. Future Perspectives

There are several questions regarding Lig3 function that will require in-depth study in the near future. A certainly not exhaustive, and yet indicative, list follows:

1. As Lig1 knockout DT40 cells proliferate normally, it must be assumed that Lig3 is recruited to DNA replication sites as effectively as Lig1. How is this achieved and how do cells chose between the two DNA ligases?
2. It has been reported that Lig1 and Lig3 have a significant contribution to translocation formation in rodent cell systems [59,60,61]. In human cells, classical NHEJ, and thus Lig4, has a bulk contribution to chromosome translocation formation initiated by DSB induced by designer nucleases [62]. However, chromosome translocations generated in human cells exposed during the G2-phase to IR, which induces DSB randomly distributed throughout the genome, largely rely on Lig1 and Lig3 as is the case in rodent cells [63]. This raises the question of the evolutionary significance of Lig3 in chromosomal translocation formation, and thus in the development of cancer.
3. Why mitochondria are served in vertebrates preferentially by Lig3 over Lig1, and why did Lig1 lose this duty during evolution?
4. As Xrcc1 is not necessary for the mitochondrial functions of Lig3, it can be inferred that Lig3 can also function without this cofactor. This raises the question as to whether certain aspects of the nuclear functions of Lig3 are also Xrcc1-independent.
5. Lig3 may have important partners in addition to Xrcc1. Indeed, Lig3 may change partners depending upon the process it is involved, and possibly also the cellular physiology. What are these partners and what is the functional significance of the corresponding interactions?
6. Although Lig2 is considered at present a biochemical artefact, the possibility should be left open that it is more than a random degradation product of Lig3: it may well be the fifth variant of Lig3 endowed with specific but as of yet uncharacterized functions.
7. Is Lig1 knockout in the mouse lethal if mediated by Cre/lox P in adult stage, where Lig3 should be able to fully compensate?

Considering the speed with which new functions and functional interactions are elucidated, we anticipate that before long answers to several of these challenging and yet highly relevant questions in the field of DNA ligases will emerge. The future is certainly bright and may hold more surprises!

## Abbreviations

BER: Base excision repair
BRCA1: breast cancer susceptibility gene 1
BRCT: BRCA1 C terminus
B-NHEJ: back up-NHEJ
C-NHEJ: classical non-homologous end-joining
Lig1: DNA ligase 1
Lig2: DNA ligase 2
Lig3: DNA ligase 3
Lig4: DNA ligase 4
IR: ionizing radiation
DSB: double strand break
HRR: homologous recombination repair
MEF: mouse embryonic fibroblasts
MTS: mitochondria target sequence
NER: nucleotide excision repair
SSBR: single strand break repair
PCNA: proliferating cell nuclear antigen
XRCC1: X-ray repair cross-complementing protein 1
